# Effect of transcutaneous posterior tibial nerve stimulation and repetitive transcranial magnetic stimulation on neurogenic overactive bladder symptoms in female patients with multiple sclerosis: The study protocol of a randomized controlled study

**DOI:** 10.3389/fneur.2022.1011502

**Published:** 2022-10-28

**Authors:** Pinar Atak Çakir, Fatma Mutluay, Lütfü Hanoğlu, Vahit Güzelburç

**Affiliations:** ^1^Department of Physiotherapy and Rehabilitation, Institute of Health Sciences, Istanbul Medipol University, Istanbul, Turkey; ^2^Neurology Department, Istanbul Medipol University, Istanbul, Turkey; ^3^Urology Department, Istanbul Medipol University Hospital, Istanbul, Turkey

**Keywords:** bladder, multiple sclerosis, neuromodulation, rTMS, PTNS

## Abstract

**Introduction:**

Neurogenic bladder is frequently seen in patients with multiple sclerosis (MS). Electrical stimulation methods (neuromodulation) can be used for patients that have persistent symptoms despite pharmacological treatment. This study aims to compare the effects of two different neuromodulation techniques used in the treatment of neurogenic bladder.

**Methods and analysis:**

This is a single-center randomized controlled trial for MS patients with neurogenic bladder. Patients determined to be eligible according to the study criteria will be randomized into two treatment groups: the transcutaneous posterior tibial nerve stimulation (PTNS) and repetitive transcranial magnetic stimulation (rTMS) groups. Each group will include eight patients. The patients will be treated for a total of 10 sessions for two consecutive weeks. The pressure-flow study will be used to compare the initial and final urodynamic results as the primary outcome. All the participants will fill in a 3-day bladder diary before and after the treatments in each group. Patients will also be asked to complete specific questionnaires for incontinence and quality of life (QOL): Overactive Bladder Questionnaire-V8 score (OAB-V8), Incontinence Severity Index (ISI), Incontinence Quality of Life Scale score (I-QOL), International Incontinence Questionnaire (ICIQ-SF) score, and International Consultation on Incontinence Questionnaire-Female Lower Urinary Tract Symptoms (ICIQ-FLUTS) score) as the secondary outcomes.

**Ethics and dissemination:**

An ethical approval number was obtained from the Non-Invasive Clinical Research Ethics Committee of Istanbul Medipol University (ethical approval number: 768). Support was received within the scope of the Istanbul Medipol University Scientific Research Project with project number 2020—2017. The result of this study will be published in a peer-reviewed journal.

**Trial registration:**

NCT05312138.

## Introduction

Multiple sclerosis (MS) is an inflammatory-demyelinating autoimmune disease of the central nervous system, characterized by inflammation, demyelination, axonal loss, and gliosis. The incidence of MS varies between 5 and 200 per 100,000. The disease affects women more than men, and its incidence is higher between the ages of 20 and 40 years (1).

Due to the heterogeneity of MS symptoms and severity, each patient is affected differently. Bladder dysfunction is one of the most bothersome symptoms of MS. Bladder dysfunction not only impairs social, professional, and sexual life activities but also leads to psychological problems, thereby negatively affecting the quality of life (QOL) of an individual (2). The frequency of bladder dysfunction in patients with MS is reported to be 52–97% (3). While urinary symptoms are among the initial symptoms in 10–15% of patients, 2% of patients are only present with these symptoms at the onset of the disease (4). According to the European Association of Urology (EUA) Guidelines published in 2022, 65% of MS patients with voiding dysfunction report detrusor overactivity (DO), 35% of detrusor sphincter dyssynergia (DSD), and 25% of detrusor underactivity (5).

Pharmacotherapy and clean intermittent catheterization constitute the first-line treatment for lower urinary tract symptoms in MS when necessary. In the literature, due to the side effects of antimuscarinic use and the lack of motivation and skill in terms of catheterization, it has been reported that electrical nerve stimulation therapy (neuromodulation) and behavior change methods (pelvic floor muscle exercise, bladder training, and keeping a urinary diary) are also used as second-line treatment options. In recent years, electrical nerve stimulation methods have started to be used in the treatment of urinary symptoms that cannot be resolved by behavioral methods in patients with neurological disorders (6).

Repetitive transcranial magnetic stimulation (rTMS) is one of the electrical nerve stimulation methods used to activate the natural electricity of the brain by creating a magnetic field without directly applying electricity to the brain. Studies have reported that rTMS therapy can be used to improve cognitive and motor functions, reduce spasticity, and treat lower urinary dysfunction (7). Although the use of rTMS in clinical settings is not yet common, it has been shown to have a very significant effect on the restoration of bladder function. In the literature, it is suggested that rTMS improves symptoms in the storage phase, increases detrusor pressure during voiding, and reduces residual urine (8).

Another electrical nerve stimulation method, posterior tibial nerve stimulation (PTNS), is a neuromodulation therapy in which the sacral nerve is stimulated through the tibial nerve in the lower part of the knee. The electrical stimulation of the posterior tibial nerve stimulates the sacral voiding center (S2–S4) *via* the sacral nerve plexus (9). Although a clear protocol for the treatment of PTNS has not yet been specified in the literature, it is reported that at least 10 sessions may be sufficient to see its benefits (10).

In this clinical study, we aim to investigate the effects of different neuromodulation methods to reduce bladder dysfunction symptoms in patients with MS. We consider that the non-invasive rTMS and PTNS treatment methods will lead to an improvement in the storage function and continence of these patients. We also aim to expand the use of these protocols for future studies.

## Methods

### Study design

This is a single-center randomized controlled trial. Informed consent will be obtained from all patients participating in the study. Starting from June 1, 2021, the first eight patients were randomized into the PTNS group and the last eight patients into the rTMS group. For randomization; eight yellow and eight blue small balls were placed in a box. The patients were asked to choose one ball. Those who chose the yellow ball were included in the PTNS group, and those who chose the blue ball were included in the rTMS group. Outcome measures will be evaluated on the data collected 3 days before and 3 days after treatment. Data analysis has not yet been performed.

### Participants

Female patients with MS will be enrolled from the Neurology Clinic of Istanbul Medipol Mega University Hospital. Patients aged 18–65 years with an Expanded Disability Status Score (EDSS) of <7.0 will be screened. All MS types, including those with relapse, were included in the study. Patients with neurogenic active bladder due to MS who have not responded to any pharmacological treatment “that is, patients who have been on antimuscarinic therapy for at least 6 months but whose incontinence continues and who cannot achieve adequate continence using a different antimuscarinic” and agree to participate in the study, will be included in the sample. According to the International Continence Society (ICS) classification, we used storage symptoms, such as urge, incontinence, nocturia, and increased voiding frequency as diagnostic criteria to define neurogenic overactive bladder (11). Patients will be asked to stop taking any pharmacotherapies for incontinence 1 month before their tests and the questionnaires. Patients with urinary tract infections, those with a diagnosis of diabetes mellitus, those using diuretic drugs or clean intermittent catheterization, and those with a history of different urological diseases will be excluded from the study. In addition, considering the contraindications for electrical stimulation, patients using a cardiac or brain pacemaker and those with epilepsy will not be included in the sample. The inclusion and exclusion criteria are detailed in Table 1.

**Table 1:** Inclusion and exclusion criteria.

**Inclusion**	**Exclusion**
18–65 years Female Voluntariness Neurogenic overactive bladder due to MS EDSS < 7.0 No response to pharmacological treatment	Urinary tract infection Diabetes mellitus Using diuretics Using clean intermittent catheterization History of other urological disease Contraindications for electrical stimulation (cardiac or brain pacemaker and epilepsy)

### Power analysis

The urodynamic test results were selected as the primary outcome. Power analysis was performed using G^*^Power v. 3.1.9.7 software package. Examining the sample size in similar studies (12), the impact power effect was accepted as 2.166667 and type I error as 0.05. As a result, the sample size was determined as eight individuals for each group to achieve a power rate of 95%.

### Interventions

#### Group 1 (PTNS)

The patient will be placed in the supine position with the medial malleolus pointing upwards, and the intervention will be performed on dry and clean skin using the TenStem device, which creates an adjustable current between 0.5 and 10 mA at a frequency of 10 Hz and with a duration of 200 μs. One of the adhesive electrodes in the device will be attached 5 cm above the medial malleus and the other electrode below the medial malleus. After turning the device on, the patient's response will be monitored, and the current will be increased according to each patient's tolerance. The patients will be informed that the current applied is harmless to their body, and this is a painless treatment method. The intervention will continue for a total of 10 sessions over 2 consecutive weeks. Each treatment session will last 20 min a day for 5 days a week. In the literature, PTNS has been reported as an effective, well-tolerated, and safe method in the management of neurogenic bladder in patients with MS (10).

#### Group 2 (rTMS)

This intervention will be performed with the patient sitting in a comfortable position. Using the PowerMAG device, which has a 70 mm figure-of-eight coil and internal cooling, 10 sessions will be undertaken over two consecutive weeks. In rTMS protocols, it is reported that high-frequency applications (≥5 Hz) increase cortical excitability while low-frequency applications (≤ 1 Hz) decrease cortical excitability (13). To increase cortical excitability, 5-Hz frequency will be used, taking previous studies as a reference. Each treatment session will last 20 min. A total of 1,000 pulses will be applied, with the number of stimuli being 50. In the literature, it has been reported that increased corticospinal tract excitability improves detrusor contraction (14).

### Outcome measures

Data will be collected at the beginning of treatment and after the end of 10 sessions of treatment. The urodynamic test, which was selected as the primary outcome, will be performed to measure the vesical and intraabdominal pressures during the filling phase (cystometry) along with the assessment of detrusor basal pressure, compliance, uninhibited contraction pressure, leak point pressures, and maximum cystometric capacity. Maximum cystometric capacity [MCC (ml)], the detrusor pressure at maximum flow rate [Pdet.Qmax (cm H_2_0)], and maximum flow rate [Qmax (ml/s)] urodynamic parameters will be evaluated. Additionally, during the pressure-flow study, flow patterns, voiding pressures, flow rates will be measured. If the patient is unable to pass urine in the presence of a vesical catheter, the patient will be asked for a free-flowmetry at the end of the urodynamic study. The urodynamic examination allows for an objective evaluation and is essential for diagnosis (15). Before all urodynamic studies, the presence of infection will be investigated using urine culture analysis in each patient.

To evaluate secondary outcomes, the data to be obtained from initial and final 72-h voiding diaries, the Overactive Bladder Questionnaire-V8 (OAB-V8), the Incontinence Severity Index (ISI), the Incontinence Quality of Life (I-QOL) questionnaire, the International Incontinence Questionnaire (ICIQ-SF), and the International Consultation on Incontinence Questionnaire-Female Lower Urinary Tract Symptoms (ICIQ-FLUTS) will be used. Considering that there may be question differences between the scales, we used a large number of scales to evaluate the patients in more detail.

The Overactive Bladder Questionnaire-V8 measures the severity of patient complaints through eight items rated on a five-point scale: not at all (0), a little bit (1), somewhat (2); quite a bit (3); a good deal (4), and a very great deal (5). The total score varies between 0 and 40 (16). The ISI consists of two questions, and the total score varies between 1 and 12, obtained by multiplying the frequency of urinary incontinence and the amount of urinary leakage (17). I-QOL comprises 22 questions presented under three subscales: limiting behavior, psychosocial impact, and social embarrassment. All the items are based on a five-point Likert type (1 = a lot, 2 = quite a bit, 3 = moderate, 4 = a little, and 5 = not at all), and these Likert types are recalculated to take a value between 0 and 100 points to better understand the total score calculated. Higher scores indicate a better quality of life (18). In clinical practice, a 3- or 7-day voiding diary is recommended (19). In the current study, a 3-day voiding diary will be used as a reliable and valid evaluation method to present the information given by the patients in an objective way. ICIQ-SF measures the extent to which urinary incontinence affects the quality of daily life (20). It is used to determine the severity, frequency, and type of incontinence. At last, ICIQ-FLUTS is a scale used to evaluate female lower urinary tract symptoms and quality of life. It is a method of evaluating the level and effect of urinary symptoms using a short but comprehensive instrument (21).

### Data analysis

The Statistical Package for Social Science (SPSS) 28.0 program will be used in the analysis. In the descriptive statistics of the data, mean, standard deviation, median, lowest, highest, frequency, and ratio values will be calculated. The distribution of variables will be measured with the Kolmogorov–Smirnov test. An independent sample *T*–test and the Mann–Whitney *U*-test will be used in the analysis of quantitative independent data, and the paired sample *T*–test and the Wilcoxon test will be used in the analysis of dependent quantitative data. In the analysis of qualitative independent data, a chi-square test and the Fischer test will be used when the chi-square test conditions are not met. The significance value will be accepted as *p* < 0.05.

### Ethics and dissemination

This study protocol was prepared in accordance with the ethical standards described in the 2013 version of the Declaration of Helsinki. Before the clinical trial began, the study protocol was reviewed by the Non-Invasive Clinical Research Ethics Committee of Istanbul Medipol University (ethical approval number: 768). The ethics committee approved the study protocol and the informed consent form. The study was also registered at ClinicalTrials.gov. Written and signed informed consent will be obtained from all the participants prior to inclusion in the study. The results of the study will be published in scientific journals and will be presented at relevant conferences.

## Discussion

Patients with MS experience adverse conditions, such as sleep disturbance, fatigue, urinary tract infection, low self-esteem, and mood disorders due to lower urinary system dysfunction. Studies have reported that the recommended treatment should have the least side effects (22). For this reason, we searched for neuromodulation methods that were reported to have the least side effects in the literature.

Neuromodulation techniques are frequently used in the rehabilitation of incontinent patients with MS. In a study by Abboud et al. it was reported that neuromodulation interventions were performed as reliable methods in the treatment of urinary dysfunction in patients with MS (23). In the literature, among neuromodulation methods, deep brain stimulation, transcranial magnetic stimulation, PTNS, sacral neuromodulation, and spinal cord stimulation are generally recommended for the treatment of bladder dysfunction. It has been shown that transcranial magnetic stimulation is more successful in the treatment of spasticity and bladder dysfunction, while PTNS provides better outcomes in terms of improving bladder dysfunction and quality of life. However, the superiority of these methods over each other remains controversial.

We reviewed all studies investigating the treatment of PTNS in patients with MS. We found evidence of a consistent positive effect on lower urinary tract symptoms, some advantages over conventional pharmacotherapy, and supporting the efficacy of PTNS in the long term. Previous studies have shown that optimal recovery can be achieved with 2 weeks of treatment, which appears to be both cost-effective and symptom-effective. However, studies evaluating its effectiveness on urodynamic parameters have found conflicting results, and improvement has been found to be more subjective (24). We think that this inconsistency may be related to the application of very different treatment protocols and the heterogeneous disease process of the MS population.

Previous studies have shown that excitatory rTMS increases corticospinal tract excitability, facilitating detrusor contraction, or urethral sphincter relaxation rather than increasing sphincter muscle activity. With rTMS, it was tried to increase the motor output and to develop control over the pontine micturition center. Neuroimaging studies have confirmed that rTMS causes changes at the subcortical level (25). The available literature is not sufficient to see definitive results.

When existing literature studies are examined, while many publications have investigated the effect of PTNS, there is only one study on transcranial magnetic stimulation, which was conducted back in 2007 and presented successful results and recommended this intervention (12). Considering these limitations in the literature, the original value of our study will be to objectively compare the effects of transcutaneous PTNS and rTMS.

In conclusion, we aim that this study will provide high-grade evidence to determine whether either transcutaneous PTNS or rTMS is superior to the other in the treatment of neurogenic bladder.

### Trial status

This study is currently ongoing. The study began in June 2021, and the first eight patients have been enrolled. The projected end date is December 2022.

### Strengths and limitations of this study

✓ This is the first randomized controlled trial to compare two different neuromodulation techniques in urological treatment.
✓ In total, 10 sessions of Transcutaneous Posterior Tibial Nerve Stimulation will be applied.
✓ The results of short treatment sessions will be investigated.
✓ Objective result measurement will be performed with urodynamics.
✓ Inclusion of only female patients may have limitations.

## Data availability statement

The original contributions presented in the study are included in the article/supplementary material, further inquiries can be directed to the corresponding author.

## Ethics statement

The studies involving human participants were reviewed and approved by Istanbul Medipol University Non-Invasive Clinical Research Ethics Committee. The patients/participants provided their written informed consent to participate in this study.

## Author contributions

PA was responsible for protocol development and handwriting. FM was the coordinator of this study. LH contributed to the collection of the data. VG played a role in ensuring the integrity of the data and the accuracy of the data analysis. All authors read and approved the final manuscript.

## Conflict of interest

The authors declare that the research was conducted in the absence of any commercial or financial relationships that could be construed as a potential conflict of interest.

## Publisher's note

All claims expressed in this article are solely those of the authors and do not necessarily represent those of their affiliated organizations, or those of the publisher, the editors and the reviewers. Any product that may be evaluated in this article, or claim that may be made by its manufacturer, is not guaranteed or endorsed by the publisher.
